# Minimum and Maximum Pattern-Based Self-Organized Feature Engineering: Fibromyalgia Detection Using Electrocardiogram Signals

**DOI:** 10.3390/diagnostics14232708

**Published:** 2024-11-30

**Authors:** Veysel Yusuf Cambay, Abdul Hafeez Baig, Emrah Aydemir, Turker Tuncer, Sengul Dogan

**Affiliations:** 1 Department of Digital Forensics Engineering, Technology Faculty, Firat University, Elazig 23119, Turkey; 231144201@firat.edu.tr (V.Y.C.); turkertuncer@firat.edu.tr (T.T.); 2 Department of Electrical and Electronics Engineering, Faculty of Engineering and Architecture, Mus Alparslan University, Mus 49250, Turkey; 3 School of Management and Enterprise, University of Southern Queensland, Toowoomba, QLD 4350, Australia; abdul.hafeez-baig@unisq.edu.au; 4 Department of Management Information Systems, Management Faculty, Sakarya University, Sakarya 54050, Turkey; emrahaydemir@sakarya.edu.tr

**Keywords:** MinMaxPat, feature engineering, ECG fibromyalgia detection, machine learning

## Abstract

Background: The primary objective of this research is to propose a new, simple, and effective feature extraction function and to investigate its classification ability using electrocardiogram (ECG) signals. Methods: In this research, we present a new and simple feature extraction function named the minimum and maximum pattern (MinMaxPat). In the proposed MinMaxPat, the signal is divided into overlapping blocks with a length of 16, and the indexes of the minimum and maximum values are identified. Then, using the computed indices, a feature map is calculated in base 16, and the histogram of the generated map is extracted to obtain the feature vector. The length of the generated feature vector is 256. To evaluate the classification ability of this feature extraction function, we present a new feature engineering model with three main phases: (i) feature extraction using MinMaxPat, (ii) cumulative weight-based iterative neighborhood component analysis (CWINCA)-based feature selection, and (iii) classification using a t-algorithm-based k-nearest neighbors (tkNN) classifier. Results: To obtain results, we applied the proposed MinMaxPat-based feature engineering model to a publicly available ECG fibromyalgia dataset. Using this dataset, three cases were analyzed, and the proposed MinMaxPat-based model achieved over 80% classification accuracy with both leave-one-record-out (LORO) cross-validation (CV) and 10-fold CV. Conclusions: These results clearly demonstrate that this simple model achieved high classification performance. Therefore, this model is surprisingly effective for ECG signal classification.

## 1. Introduction

Fibromyalgia is a chronic pain syndrome associated with the central nervous system [1,2] and is characterized by widespread sleep disorders, musculoskeletal pain, cognitive dysfunction, and fatigue [3]. Patients experience chronic widespread pain in different areas of their bodies, and extreme sensitivity can be seen, especially at certain points. These tender points play an important role in the diagnosis of fibromyalgia [4]. Fatigue and sleep disorders are common in fibromyalgia patients and are often severe enough to limit daily activities [5,6]. Cognitive dysfunction, known as “fibro fog”, is also frequent [7]. There is no specific laboratory test for diagnosing fibromyalgia; however, clinical evaluation and physical examination are essential [8]. The American College of Rheumatology (ACR) criteria use the Widespread Pain Index (WPI) and Symptom Severity Scale (SSS) to assess pain and symptom severity [9,10]. Sleep Polygraphy (Polysomnography) may be used to evaluate sleep disorders, while laboratory tests such as Complete Blood Count (CBC), Thyroid Function Tests, and Erythrocyte Sedimentation Rate (ESR) help exclude inflammatory and autoimmune diseases [11].

Treatment for fibromyalgia includes medication, physical therapy, and psychotherapy [12]. Common medications are Tricyclic Antidepressants (TCAa), Serotonin–Norepinephrine Reuptake Inhibitors (SNRIs), Gabapentinoids, and Nonsteroidal Anti-Inflammatory Drugs (NSAIDs) [13]. Physical therapy and exercise help reduce pain by strengthening muscles. Cognitive–Behavioral Therapy (CBT) and Mindfulness-Based Stress Reduction (MBSR) are used to manage pain [14]. Artificial intelligence (AI) tools like machine learning (ML) and deep learning (DL) also help doctors understand symptoms and create personalized treatments [15].

### 1.1. Literature Review

Some studies on fibromyalgia in the literature are presented in Table 1.

### 1.2. Literature Gaps

The detected literature gaps highlight that the minimum and maximum statistical moments have been utilized in both deep learning (DL) and feature engineering (FE) [29]. In FE models, these moments are typically employed to generate statistical features or normalization [30], whereas in DL models, they are used for pooling functions and normalization [31]. However, no existing descriptor specifically based on these moments has been identified in the literature, as confirmed by our knowledge and review. Additionally, while DL models have been extensively used to achieve high classification performance [32], they are inherently complex and often involve exponential time complexity [33,34]. Consequently, training such models on local devices requires expensive hardware, such as graphical processing units (GPUs) [35].

### 1.3. Motivation and Our Model

The major motivation of this research is to fill the existing gaps in the literature. Therefore, we have proposed a new feature extraction function based on simple mathematical definitions, specifically using two basic statistical moments: minimum and maximum. By deploying these moments, we developed a new feature extraction function, which serves as a one-dimensional descriptor. This feature extraction method uses a local feature extraction approximation and is named MinMaxPat (minimum and maximum pattern).

To investigate the classification performance of the MinMaxPat, we proposed a new feature engineering (FE) model. This model consists of three main phases: (i) MinMaxPat-based feature extraction, (ii) feature selection using cumulative weighted iterative neighborhood component analysis (CWINCA), and (iii) classification using a t-algorithm-based k-nearest neighbors (tkNN) [36] classifier. By employing this strategy, a lightweight FE model was created, as the methods used in the MinMaxPat-based FE model have linear time complexity. Thus, the proposed model is both lightweight and efficient for signal classification.

In this research, we tested a new coding schema using the well-known statistical moments—maximum and minimum—and the proposed MinMaxPat. Our goal was to develop a new signal descriptor similar to the local binary pattern (LBP) [37]. In the testing phase, our proposed MinMaxPat surprisingly achieved high classification performance. Therefore, a new feature engineering model was developed by integrating the CWINCA and tkNN methods. The CWINCA feature selection method identified the most informative features from the 256 generated features, while tkNN, an ensemble classifier, generated the classification results. Both CWINCA and tkNN methods are self-organized, making the proposed FE model a self-organized feature engineering (SOFE) model.

### 1.4. Innovations and Contributions

A novel feature extraction function, termed MinMaxPat, has been developed. This function serves as the foundation for a new self-organized feature engineering (SOFE) model, which is also introduced in this study.

The main motivation behind this work is to design a simple yet effective descriptor similar to the local binary pattern (LBP). The proposed MinMaxPat leverages minimum and maximum statistical moments along with a base-16 coding method to generate features. Additionally, it employs histogram-based feature extraction, making it a significant contribution to the feature extraction research area.

Using MinMaxPat, a new SOFE model has been presented with a streamlined architecture consisting of three phases: (i) feature extraction, (ii) feature selection, and (iii) classification. Features are extracted using MinMaxPat, while the feature selection and classification phases employ two self-organized methods, ensuring efficiency and adaptability. This integrated approach establishes a new paradigm in SOFE research and highlights the potential of simple architectures for achieving high-performance results.

## 2. Materials and Methods

### 2.1. Material

A publicly available ECG fibromyalgia dataset [17] was used in this research. This dataset was collected from participants during sleep stage 2 and sleep stage 3, consisting of 139 records. The collected ECG signals are single-lead signals, and the sampling frequency of these ECG signals is 512 Hz. We divided the ECG signals into segments, each with a length of 15 s. Therefore, each ECG segment contains 7680 (=15 × 512) values, which we used as an array of length 7680. There are two main classes in this dataset, (i) fibromyalgia and (ii) control, as the objective of this dataset is to detect differences between healthy and fibromyalgia ECG signals.

Using these ECG signals, we created three cases, and the features of these cases are tabulated in Table 2.

In order to obtain results from these ECG signals, we used both 10-fold cross-validation (CV) and leave-one-record-out (LORO) CV to provide reliable results.

### 2.2. The Presented MinMaxPat-Based SOFE

In this research, we propose a new SOFE model based on the MinMaxPat method. This model consists of three main phases, two of which are self-organized. The phases are as follows: (1) MinMaxPat-based feature extraction, (2) CWINCA-based feature selection, and (3) tkNN-based classification.

**Figure 1:**
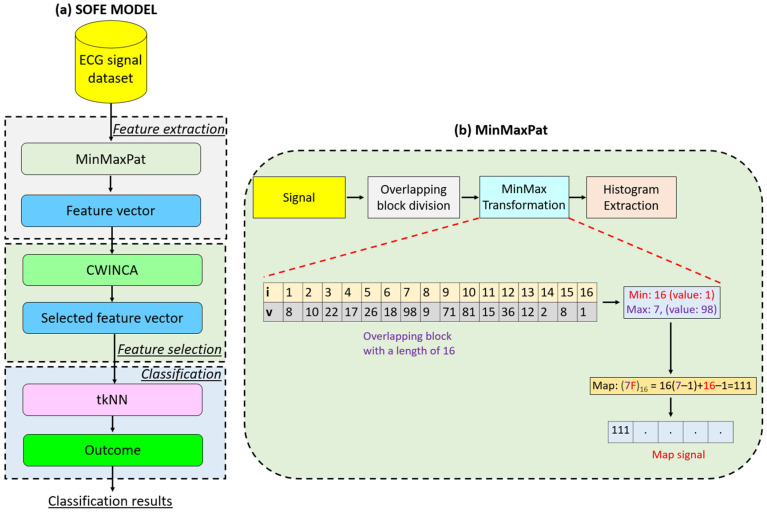
Graphical overview of (**a**) the proposed MinMaxPat-based SOFE model and (**b**) the presented MinMaxPat.

To select the most informative features from these 256 features, the CWINCA feature selector was used. By utilizing the selected features as input for the tkNN classifier, the classification results were generated. To better explain the proposed MinMaxPat-based SOFE model, a graphical depiction is shown in Figure 1.

The details of the proposed SOFE model are provided step by step below.

Step 1: Apply MinMaxPat to generate 256 features from each ECG signal. The details of the MinMaxPat method are explained below.

In Figure 1b, we demonstrate a block diagram of the proposed MinMaxPat along with a numerical example. The steps of this descriptor are also provided below.

Step 1.1. Divide the signal into overlapping blocks with a length of 16.

(1) $$block^{i}\left(j\right)=ECG\left(i+j-1\right),\;i\in \left\{1,2,\ldots ,Len-15\right\},j\in \left\{1,2,\ldots ,16\right\}$$

where block: overlapping block with a length of 16; ECG: ECG signal; and Len: length of the signal.

Step 1.2. Find indexes of the minimum and maximum values of the block.

(2) $$mini\left(i\right)=argmin(block^{i})$$

(3) $$maxi\left(i\right)=argmax(block^{i})$$

Here, mini: minimum index and maxi: maximum index of the block. Moreover, the argmin(.) and argmax(.) functions detect indices of the minimum and maximum values.

Step 1.3. Calculate the feature map value.

(4) $$map\left(i\right)=16\left(maxi\left(i\right)-1\right)+\left(mini\left(i\right)-1\right)$$

where map: feature map signal coded as base16.

Step 1.4. Extract the histogram of the feature map signal to obtain the feature vector.

(5) $$feat\left(d,1:256\right)=\xi \left(map\right),\;d\in \left\{1,2,\ldots ,NoS\right\}$$

Here, feat: features; ξ(.): histogram extraction function; NoS: number of sample.

The given Steps 1.1–1.4 are defined as the proposed MinMaxPat feature extraction function.

Step 2. Select the best features by deploying the CWINCA feature selector.

The CWINCA function used is a self-organized feature selector. By deploying cumulative weights, we determined the start and stop indexes for the loop of the INCA [38]. Subsequently, iterative feature selection was applied using the INCA feature selector, and the best feature vectors were selected automatically. In this regard, CWINCA functions as a self-organized feature selector. The steps of this feature selector are defined below.

(6) $$\left[w,id\right]=NCA(feat,y)$$

where w: weight of the features; id: indices of the sorted features obtained by computing feature weights; NCA(.): NCA feature selection function; and y: actual output.

Step 2.2. Compute start and stop values by deploying cumulative weight computation. Herein, in order to compute the start and stop values, we have used 0.80 and 0.99 threshold values.

(7) start=CW(w,id,0.80)

(8) stop=CW(w,id,0.99)

Herein, start: start value of the loop; stop: stop value of the loop; and $CW\left(.\right):$ cumulative weight computation function.

Step 2.3. Select features iteratively and compute the loss values of the selected features.

(9) $$fs^{r-start+1}\left(d,i\right)=feat\left(d,ind\left(i\right)\right),\;i\in \left\{1,2,\ldots ,r\right\},\;r\in \left\{start,start+1,\ldots ,stop\right\}$$

(10) $$loss\left(r-start+1\right)=C(fs^{r-start+1},y)$$

where fs: selected feature vector; loss: the misclassification rate; and C: classifier.

Step 2.4. Choose the final feature vector by deploying the greedy algorithm.

(11) $$ca\left(i\right)=\varphi (fs^{r-start+1},y)$$

(12) id=argmax(ca)

(13) $$SF={fs}^{id}$$

Here, ca: classification accuracy; φ(.): classification accuracy calculation function; id: indices of the maximum classification accuracy; and SF: the final selected feature vector.

Step 3: Classify the selected features by deploying the tkNN classifier. The tkNN classifier is an ensemble and self-organized classifier. In this classifier, iterative parameter changes are applied, and parameter-based outcomes are generated. Subsequently, iterative majority voting (IMV) [39] is applied to these parameter-based outcomes, and voted outcomes are produced. In the final phase of the tkNN algorithm, the best outcome (the one with the maximum classification accuracy) is selected using a greedy algorithm. In this sense, the tkNN classifier is a self-organized classifier. The steps of this classifier are as follows:

Step 3.1. Generate parameter-based outcomes by deploying iterative parameter changes and the kNN classifier.

(14) $$pot_{a}=kNN\left(SF,y,D_{i},K_{j},W_{k}\right),a\in \{1,2,\ldots ,90\}$$

(15) D∈{Cityblock,Euclidean,Cosine}

(16) K∈{1,2,…,10}

(17) D∈{Equal,Inverse,SquaredInverse}

where pot: parameter-based outcome; kNN(.): kNN classifier [40]; D: distance; K: k-values; and W: weights. We used 3 distances, 10 k values, and 3 weights. Therefore, the tkNN classifier generated 90 (=3 × 3 × 10) classifier-based outcomes.

Step 3.2. Deploy the IMV algorithm and generate 88 more voted outcomes.

(18) $$ca\left(a\right)=\varphi \left(pot_{a},y\right)$$

(19) $$ix=argsort\left(-ca\right)$$

(20) $$vot_{b}=\varpi \left(pot_{ix\left(1\right)},pot_{ix\left(2\right)},\ldots ,pot_{ix\left(b+2\right)}\right),b\in \{1,2,\ldots ,88\}$$

Here, ix: the qualified indices; vot: voted outcomes; and ϖ(.): mode function.

Step 3.3. Choose the best outcome by deploying the greedy algorithm to the generated 178 outcomes.

(21) $$ca\left(90+b\right)=\varphi (vot_{b},y)$$

(22) x=argmax(ca)

(23) $$FinalOut=\left\{\begin{array}{c} pot_{x},\;x\leq 90\\ vot_{x-90},x>90 \end{array}\right.$$

Here, x: index of the maximum classification accuracy and FinalOut: the final/ultimate outcome.

These three steps are defined as the proposed MinMaxPat-based SOFE model.

## 3. Results

In order to investigate the classification performance of the proposed MinMaxPat-based SOFE model, it was applied to an ECG fibromyalgia dataset. The dataset was downloaded, and cases were created for analysis. Subsequently, the MinMaxPat-based SOFE model was developed using MATLAB (version 2024a), employing functional programming with four key functions: (i) main, (ii) MinMaxPat, (iii) CWINCA, and (iv) tkNN.

The used feature extraction function is a basic function and the time complexity of this function is computed as O(n). Herein, n: length of the signal and O(.): big O notation. In the feature selection phase, we used an iterative feature selector called CWINCA. The time complexity of the CWINCA feature selector is equal to O(N+iK). Herein, N: time complexity coefficient of the NCA [41]; i: number of iterations; and K: time complexity of the used classifier to compute misclassification rates. The last phase is the classification phase. The tkNN classifier is an iterative classifier. Therefore, the time complexity of this classifier is equal to O(iK). In this aspect, the total time complexity of the recommended model is equal to $O\left(n+N+iK+iK\right)≅O\left(n+N+iK\right)$. This computation clearly demonstrated the linear time complexity of the recommended MinMaxPat-based SOFE model. Therefore, we used a central processing unit (CPU) mode to implement the presented MinMaxPat-based model.

The recommended MinMaxPat-based model is a parametric model, and to provide a repetition of this model, the initial parameters used in this model are given in Table 3.

By deploying the parameters listed in Table 3, the proposed MinMaxPat-based model was created.

To obtain the classification results, we used five commonly known performance evaluation metrics: (i) classification accuracy, (ii) geometric mean, (iii) precision, (iv) recall, and (v) F1-score.

**Figure 2:**
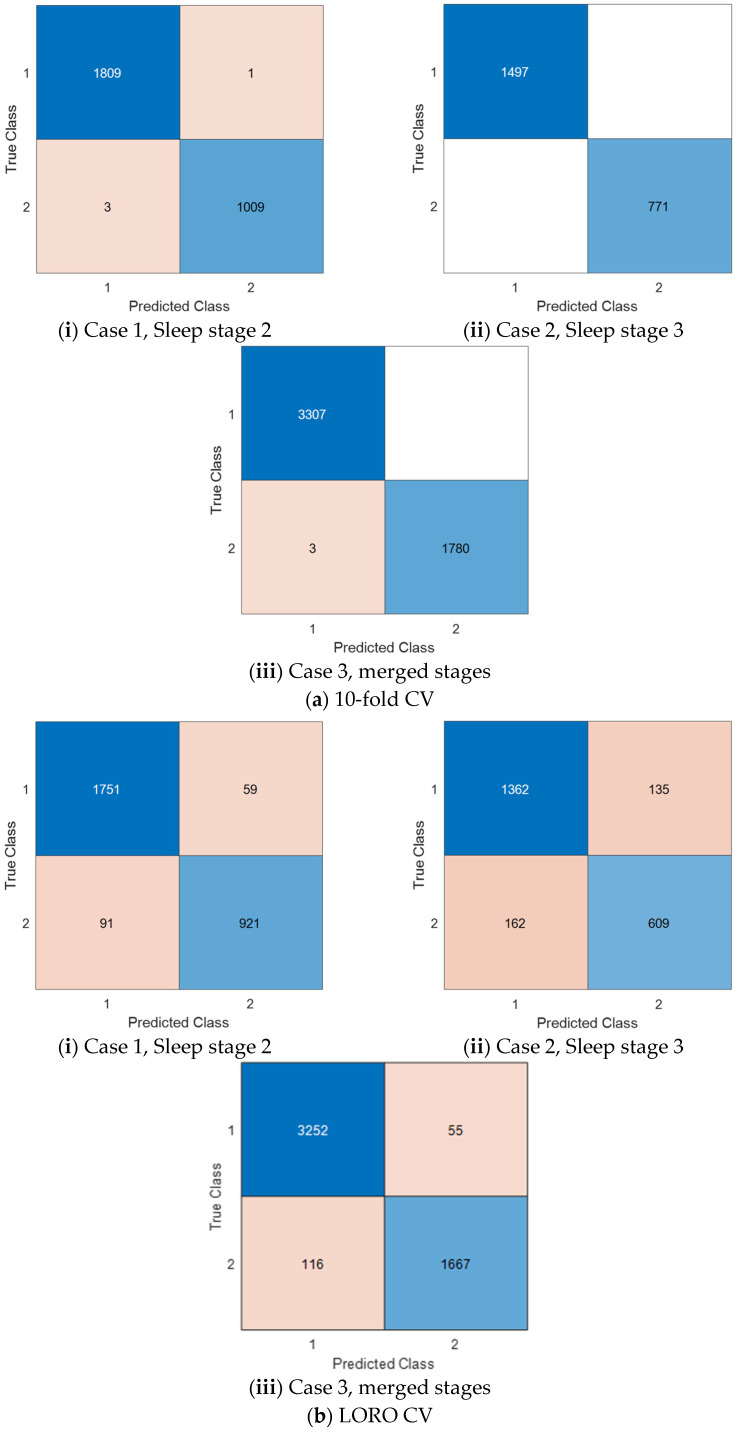
The computed confusion matrices per the cases and the used validation techniques. Herein, 1: control; 2: fibromyalgia. (**a**) 10-fold CV; (**b**) LORO CV.

Moreover, we applied three cases and used two validation techniques: 10-fold CV and LORO CV. To compute the results, we used confusion matrices for these cases based on the validation technique. The computed confusion matrices are shown in Figure 2.

By using the given confusion matrices in Figure 2, the results of this model were computed and are listed in Table 4.

Table 4 shows that the highest classification accuracy and geometric mean were 100% for the ECG signals collected during sleep stage 3. For LORO CV, the best classification accuracy and geometric mean were 96.64% and 95.88%, respectively, in Case 3. Additionally, the proposed MinMaxPat-based model achieved over 84% classification performance for all cases using both validation techniques.

**Figure 3:**
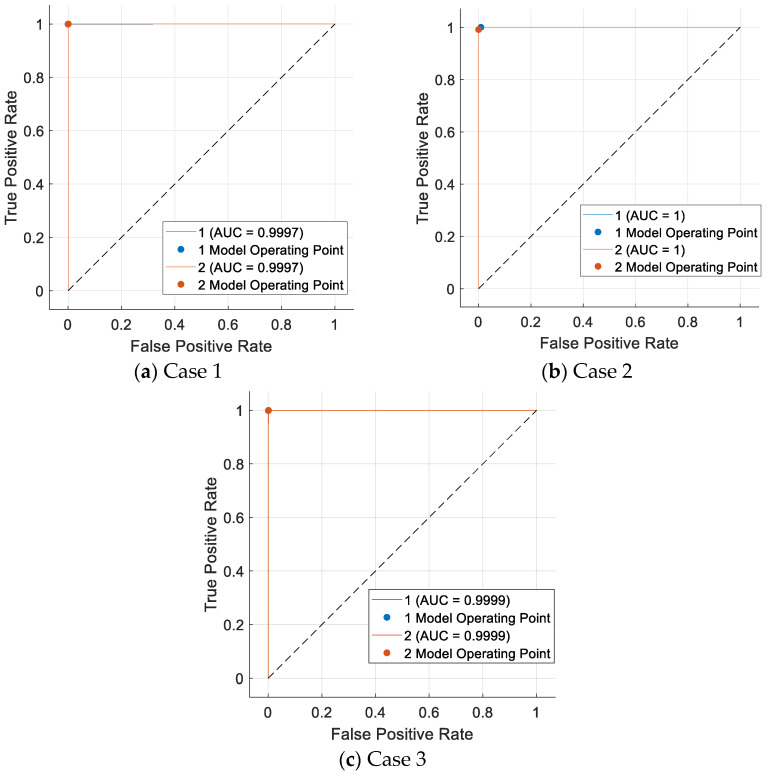
The computed ROC curves. (**a**) Case 1; (**b**) Case 2; (**c**) Case 3.

Moreover, the receiver operating characteristic (ROC) curves of the introduced model for the defined Cases 1, 2, and 3 are given in Figure 3.

Per the given ROC curves, all recommended area under curve (AUC) values are above 0.999.

The performance of the MinMaxPat-based SOFE model lies in its ability to maintain high accuracy even with few features. By selecting the most informative features (38 for Case 1, 35 for Case 2, and 31 for Case 3) using CWINCA, the model demonstrates that it can achieve high classification accuracy with minimal computational load. Furthermore, the highest accuracy rates for 10-fold CV were obtained in sleep stage 3 (Case 2), showcasing the model’s ability to effectively handle specific and challenging cases. These results clearly demonstrate the effectiveness of the MinMaxPat-based SOFE model. A discussion of the model’s results is provided in Section 4, along with a more in-depth analysis.

## 4. Discussion

In this research, we proposed a new MinMaxPat-based SOFE model to investigate the classification performance of the MinMaxPat feature extraction. We presented a simple structure for the SOFE model, which consists of three main phases. In the feature selection and classification phases, we used self-organized CWINCA and tkNN methods, making this feature engineering model self-organized.

In the feature extraction phase, we extracted 256 features from each ECG signal, and CWINCA selected the most informative 38, 35, and 31 features as the final feature vectors for Case 1, Case 2, and Case 3, respectively. At this point, the proposed model achieved high classification performance with fewer features.

**Figure 4:**
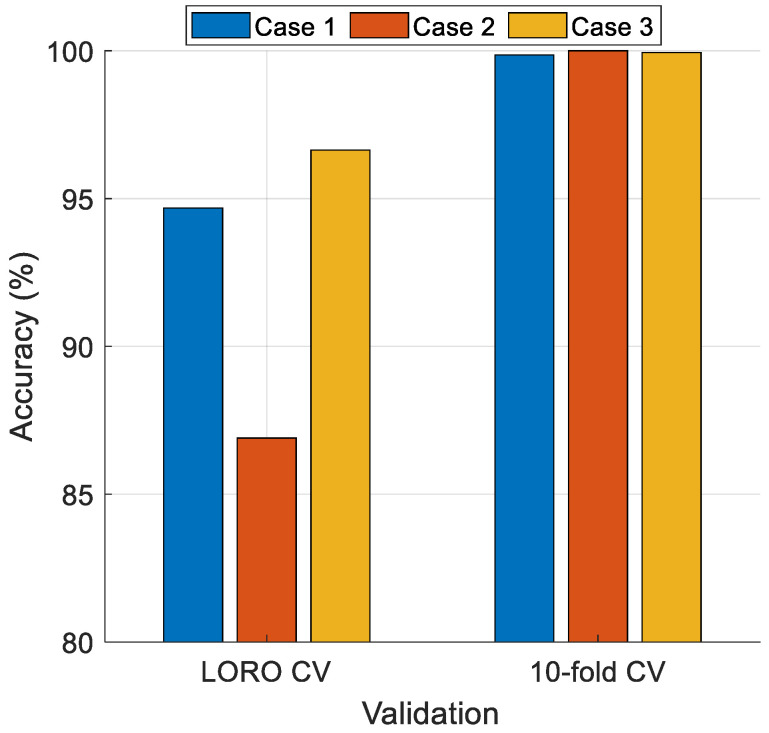
Performance comparison of the proposed model according to cases.

We also used the 10-fold CV and LORO CV techniques in the MinMaxPat-based SOFE model, and the results are demonstrated in Table 4. According to Table 4, the worst case is Case 2, with a classification accuracy of 86.90% using LORO CV. However, Case 2 performed the best using 10-fold CV, achieving 100% classification accuracy. To better understand the performance of each case, the comparative results are showcased in Figure 4.

Figure 4 shows that the best cases for LORO CV and 10-fold CV are Case 3 and Case 2, respectively. Moreover, for 10-fold CV, all cases achieved over 99% classification accuracy (see Table 4). Additionally, both Case 1 and Case 3 attained over 90% classification accuracy using both validation techniques.

**Figure 5:**
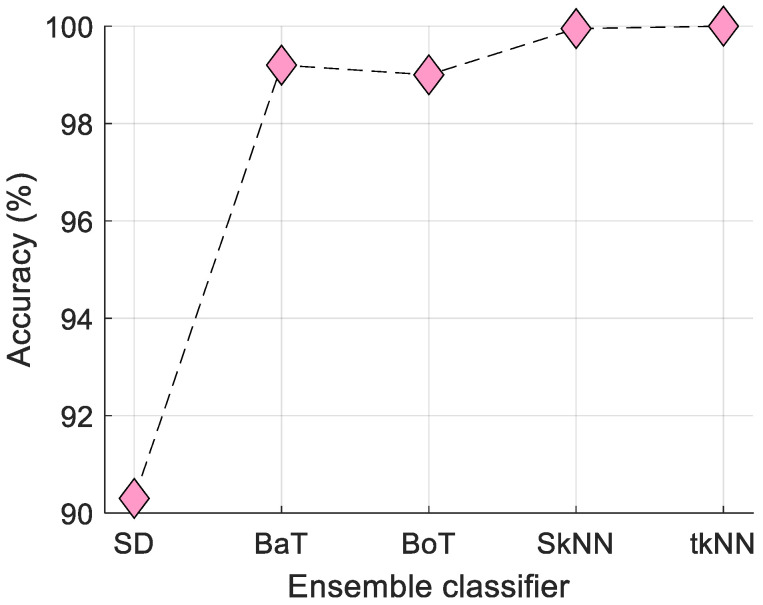
Comparative results of the ensemble classifiers on Case 2 with 10-fold CV.

The tkNN classifier used is an ensemble classifier. Therefore, we compared the classification performance of the tkNN classifier to other ensemble classifiers, namely (i) subspace discriminant (SD), (ii) subspace kNN (SkNN), (iii) bagged tree (BaT), and (iv) boosted tree (BoT). The comparative results of tkNN with these classifiers are displayed in Figure 5.

Figure 5 shows that the closest classifier to tkNN is SkNN, which achieved 99.95% classification accuracy, while tkNN achieved 100% classification accuracy. Therefore, we selected the tkNN classifier for this model.

**Figure 6:**
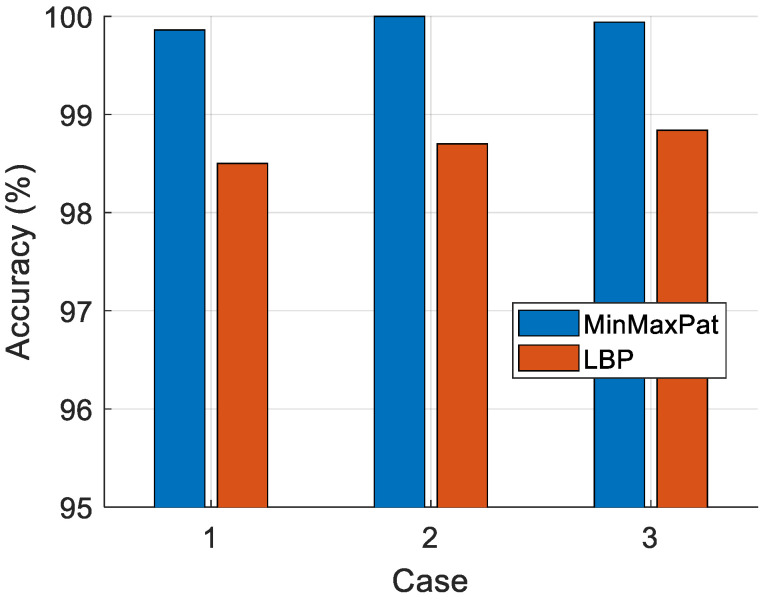
MinMaxPat versus one-dimensional LBP.

The proposed MinMaxPat is a simple feature extractor (signal descriptor), inspired by the local binary pattern (LBP). We aimed to create a more effective and simpler feature extraction function than LBP, which led to the development of MinMaxPat. To ensure a fair comparison, we used both LBP and MinMaxPat as feature extractors and conducted ablation studies. The classification results computed using 10-fold CV are also demonstrated in Figure 6.

Figure 6 clearly demonstrates that the proposed MinMaxPat achieved over 99% classification accuracy for all cases, while the LBP feature extraction function could not reach 99% accuracy in all cases.

To highlight the significance of the proposed model in the literature, we present comparative results in Table 5.

**Figure 7:**
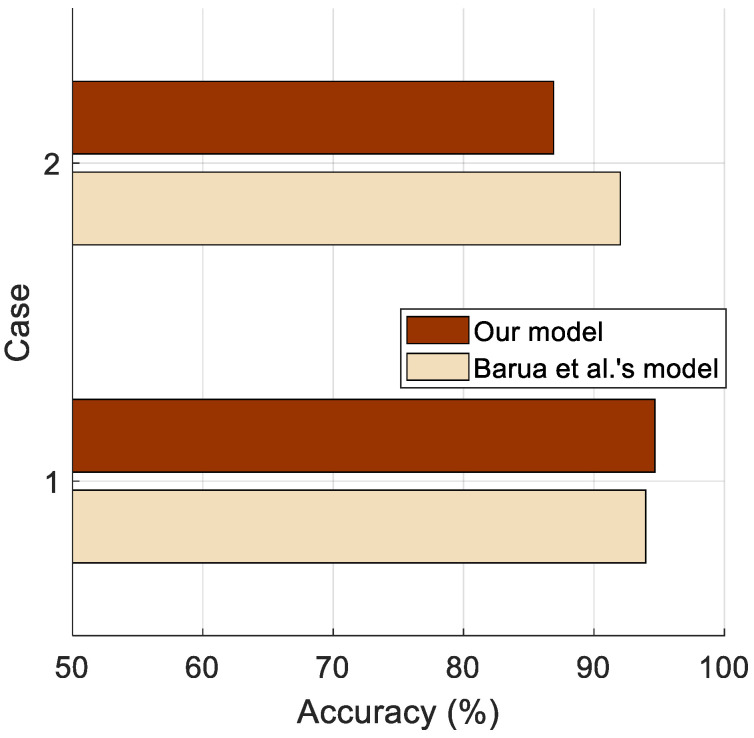
Comparative results with LORO CV (Our model and Barua et al. [42]).

The results in Table 5 indicate that the proposed model outperforms Barua et al.’s method for Case 1, achieving an accuracy of 94.68% compared to 93.97%. This clearly highlights the effectiveness of the presented MinMaxPat feature extraction approach. While Barua et al.’s method achieves higher accuracy in Case 2, their approach is significantly more complex, requiring multiple feature selectors, classifiers, and a knowledge fusion stage. In contrast, the proposed MinMaxPat-based SOFE model employs a simple three-stage process that includes MinMaxPat for feature extraction, CWINCA for feature selection, and tkNN for classification. Moreover, the comparative results are shown in Figure 7 [42].

Table 5, Figure 7, and these models’ structures demonstrate that the simplicity of the MinMaxPat-based SOFE model does not compromise its effectiveness. The proposed model offers competitive performance in Case 1, presenting an accessible and efficient approach to feature extraction and classification. Furthermore, by focusing on lightweight computation, the MinMaxPat-based SOFE model provides a practical solution for real-time fibromyalgia detection systems, advancing the field of feature engineering by balancing simplicity and performance. The introduced model’s straightforward structure allows it to be implemented in various environments, such as FPGA, and it effectively distinguishes ECG signals. Additionally, its adaptability makes it suitable for other signal structures with minimal modifications.

**Figure 8:**
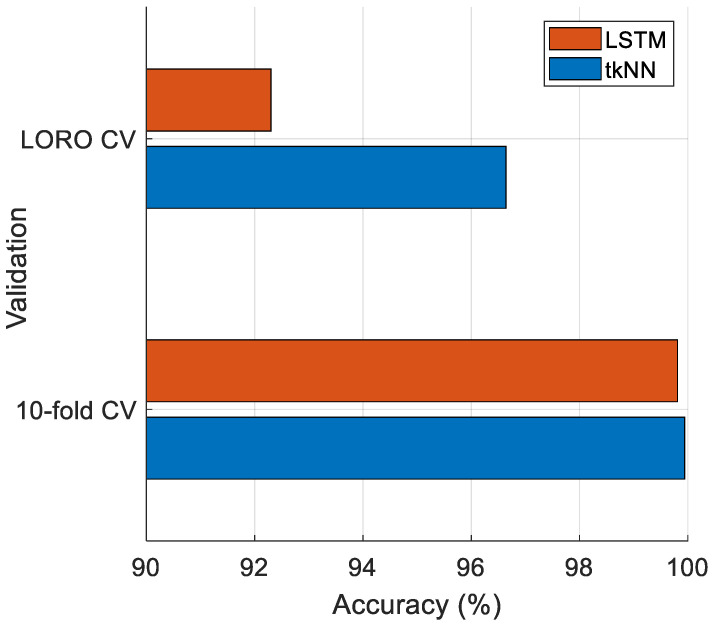
Comparison of tkNN and LSTM for Case 3 deploying 10-fold CV.

Furthermore, we used an LSTM classifier to obtain the classification results, and the selected features achieved accuracies of 99.81% and 92.30% using LSTM with 10-fold CV and LORO CV, respectively, while the tkNN classifier attained 99.84% and 96.64% classification accuracy with 10-fold CV and LORO CV, respectively. The hyperparameters for the LSTM classifier were as follows: maximum epochs: 50, number of hidden units: 100, mini-batch size: 64, initial learning rate: 0.01, gradient threshold: 1, and validation using 10-fold CV. For Case 3 (the largest case, encompassing Case 1 and Case 2), the classification results of the LSTM and tkNN classifiers are depicted in Figure 8.

Figure 8 clearly shows that the tkNN classifier achieved higher classification accuracy compared to the LSTM classifier for Case 3.

The findings of this research study are as follows: The MinMaxPat-based SOFE model achieved high classification performance across all three defined cases. This success can be attributed to the feature extraction phase, where 256 meaningful features are extracted from each ECG signal using MinMaxPat, and CWINCA selects the most informative features. The model maintained high classification accuracy with a reduced number of features, selecting 38 for Case 1, 35 for Case 2, and 31 for Case 3. The performance of the proposed model was evaluated using both 10-fold cross-validation (CV) and leave-one-record-out (LORO) CV, producing robust and reliable results with both techniques. Notably, Case 2 achieved an accuracy rate of 86.90% with LORO CV and 100% accuracy with 10-fold CV. The proposed SOFE model also demonstrated strong performance in Cases 1 and 3, with Case 3 identified as the best-performing case in both validation techniques.

The tkNN classifier used in the model was compared against ensemble classifiers such as SD, SkNN, BaT, and BoT to validate its superiority. Using 10-fold CV, tkNN achieved 100% accuracy in Case 2, outperforming its closest competitor, SkNN, which achieved 99.95% accuracy. Furthermore, the MinMaxPat feature extraction method outperformed the classification performance of the LBP feature extractor, proving that it is a simple yet effective method. When compared with existing methods in the literature, the proposed method consistently outperformed its competitors, further validating its effectiveness.

The advantages of the recommended model are given as follows. The proposed model achieved high classification performance, notably reaching 100% accuracy in Case 2 with 10-fold CV. By employing both leave-one-record-out (LORO) CV and 10-fold CV, the model produced robust and reliable results. Despite its simplicity, the MinMaxPat feature extraction function demonstrated remarkable effectiveness in feature extraction and classification tasks. With its linear time complexity, the MinMaxPat-based model is lightweight and computationally efficient. The integration of self-organized methods, specifically CWINCA for feature selection and tkNN for classification, further enhanced the model’s performance, establishing it as a self-organized feature engineering (SOFE) approach. Consistently strong performance across different cases highlights the model’s reliability and versatility.

The limitations of this research are as follows: The classification performance of the proposed MinMaxPat-based SOFE model dropped to 86.90% accuracy for sleep stage 3 when evaluated using LORO CV. To further validate and enhance the model’s performance, more diverse and larger datasets could be utilized for testing.

The future works of this research are as follows: The proposed MinMaxPat-based SOFE model can be extended to larger and more diverse datasets, including ECG data from different states (e.g., awake or exercising), to enhance its generalizability. To address the performance degradation observed in Case 2, more advanced feature selection and classification techniques, such as deep learning, can be integrated into the model, and the performance of these deep models can also be evaluated as benchmarks. Despite its simplicity, the MinMaxPat-based SOFE model is an effective FE approach with manageable time complexity, making it highly suitable for real-time fibromyalgia detection systems and a viable option for practical applications. Additionally, future models could incorporate other physiological signals, such as electromyography (EMG) or functional near-infrared spectroscopy (fNIRS), alongside ECG data to improve detection accuracy and address more complex cases. Furthermore, research efforts can focus on developing next-generation MinMaxPat-based deep learning models to advance biomedical signal classification further.

## 5. Conclusions

The presented MinMaxPat-based SOFE model has demonstrated its effectiveness in detecting fibromyalgia by analyzing ECG signals. The model utilizes MinMaxPat for feature extraction, generating 256 features from each signal, with the most informative features being selected using the CWINCA method. The tkNN classifier, integrated within the model, enabled high classification performance across multiple cases, reaching 100% classification accuracy for Case 2 with 10-fold cross-validation and 99.94% accuracy for the merged sleep stages.

When validated with LORO cross-validation, the model achieved 94.68% accuracy for Case 1 and 96.64% accuracy for the merged sleep stages. The geometric mean values were similarly high, ensuring reliable results across different validation methods. Compared to other classifiers, such as SkNN, the tkNN-based classifier outperformed its counterparts, achieving superior results in the fibromyalgia detection task.

This work also shows that the MinMaxPat feature extraction method is more effective than traditional approaches like LBP, as evidenced by its consistent classification accuracy exceeding 99%. Additionally, the model outperformed Barua et al.’s approach in Case 1, indicating the robustness of MinMaxPat as a feature extraction method in biomedical signal analysis [42].

These findings and the computed results showcase that the MinMaxPat-based SOFE model is a reliable tool for fibromyalgia detection, offering a balance between simplicity and accuracy in the classification of ECG signals.

## Figures and Tables

**Table 1:** Literature review.

Study	Data	Method	Split Ratio	Results (%)
Sabeti et al. [16]	Physiological signals	Learning using concave and convex kernels	10-fold CV	Acc: 88.38
Paul et al. [17]	Sleep EEG signals	Nonlinear dynamical features	10-fold CV	Acc: 96.15 Sen: 96.88 Spe: 95.65
Santana et al. [18]	rs-fMRI	Dynamic time warping	5-fold CV	Acc: 86.00 ROC: 93.00
Chatterjee et al. [19]	rs-fMRI	CNN	5-fold CV	Acc: 85.20 Pre: 83.00 Rec: 98.00 F1: 90.00
Fukae et al. [20]	Clinical data	CNN	80:20	Acc: 98.00
Gokcay et al. [21]	fNIRS and clinical data	Likelihood-based decision level fusion	10-fold CV 20-fold CV	Spe: 100.0 Sen: 100.0
Alves et al. [22]	Blood plasma samples	Paper spray ionization–mass spectrometry	70:30	Acc: 100.0
Robinson et al. [23]	Structural magnetic resonance imaging and self-report data	J48 decision tree	10-fold CV	Acc: 96.17
Martín-Brufau et al. [24]	Resting-state EEG recordings	Fast Fourier transform, statistical analysis	Unspecified	High discriminative capacity: 100%
Passos et al. [25]	Blood plasma samples	Chemometric analysis	70:15:15	Acc: 84.20 Sen: 89.50 Spe: 79.00
Orrù et al. [26]	Psychometric tests	J48 decision tree	10-fold CV	Acc: 88.16 AUC: 88.00 F1: 88.00
Alves et al. [27]	Blood plasma samples, clinical data	Principal component analysis	10-fold CV	Acc: 88.00 Sen: 100.0 Spe: 75.00 AUC: 87.00
Aksalli et al. [28]	Sleep EEG signals	D’hondt pooling, glucose pattern	1. 10-fold CV 2. Leave-one-record-out	1. Acc: 100.0 Pre: 100.0 Rec: 100.0 F1: 100.0 2. Acc: 99.72 Pre: 99.74 Rec: 9982 F1: 99.78

rs-fMRI: resting-state functional magnetic resonance imaging; CNN: Convolutional Neural Network; fNIRS: functional near-infrared spectroscopy.

**Table 2:** Features of the cases.

Case	Class	Number of Records	Number of Segments
1: Sleep stage 2	Control	42	1810
	Fibromyalgia	32	1012
	Total	74	2822
2: Sleep stage 3	Control	36	1497
	Fibromyalgia	29	771
	Total	65	2268
3: Merged	Control	78	3307
	Fibromyalgia	61	1783
	Total	139	5090

**Table 3:** The initial parameters of the introduced MinMaxPat-based SOFE.

Phase	Method	Parameters
Feature extraction	MinMaxPat	Input: ECG signal Number of leads: 1 Length of each overlapping blocks: 16 Base: 16 Output: 256 features
Feature selection	CWINCA	Input: Generated feature vector Loop range detector: Cumulative weights Threshold values: 0.85 for start value and 0.99 for stop value Output: Selected feature vector
Classification	tkNN	Input: Selected feature vector Distances: City block, Cosine, Euclidean k value: from 1 to 10 Weight: Squared inverse, equal, inverse Number of the parameter-based outcome: 90 Number of voted outcome: 88 Selection criteria: Maximum classification accuracy Output: tkNN-based outcomes Parameters of the IMV: Loop range: from 3 to 90

**Table 4:** The computed classification results.

Validation	Performance Evaluation Metric	Case 1: Sleep Stage 2	Case 2: Sleep Stage 3	Case 3: Merged Sleep Stage
10-fold CV	Accuracy	99.86	100	99.94
	Geometric mean	99.82	100	99.92
	Precision	99.87	100	99.95
	Recall	99.82	100	99.92
	F1-score	99.85	100	99.94
LORO CV	Accuracy	94.68	86.90	96.64
	Geometric mean	93.83	84.77	95.88
	Precision	94.52	85.61	96.68
	Recall	93.87	84.99	95.92
	F1-score	94.18	85.28	96.28

**Table 5:** Comparative results deploying LORO CV.

Research	Method	Accuracy (%)
Barua et al. [42]	Multiple filter-based discrete wavelet transform, quantum inspired local binary pattern (3LBP), Chi2- and NCA-based feature selection, kNN and support vector machine classifiers, IMV-based information fusion	Case 1: 93.97 Case 2: 92.02
Our model	MinMaxPat, CWINCA, tkNN	Case 1: 94.68 Case 2: 86.90

## Data Availability

The authors are committed to making the data available if requested by the Journal.
